# Interaction Matters: The Effect of Touching the Social Robot PARO on Pain and Stress is Stronger When Turned ON vs. OFF

**DOI:** 10.3389/frobt.2022.926185

**Published:** 2022-07-08

**Authors:** Nirit Geva, Netta Hermoni, Shelly Levy-Tzedek

**Affiliations:** ^1^ Recanati School for Community Health Professions, Department of Physical Therapy, Ben-Gurion University of the Negev, Beer-Sheva, Israel; ^2^ Department of Biomedical Engineering, Ben-Gurion University of the Negev, Beer-Sheva, Israel; ^3^ Zlotowski Center for Neuroscience, Ben-Gurion University of the Negev, Beer-Sheva, Israel; ^4^ Freiburg Institute for Advanced Studies (FRIAS), University of Freiburg, Freiburg, Germany

**Keywords:** pain, human-robot interaction, stress, interactivity, sociality, touch

## Abstract

Social touch between humans, as well as between humans and animals, was previously found to reduce pain and stress. We previously reported that touching a social robot can also induce a reduction in pain ratings. However, it is unclear if the effect that touching a robot has on pain perception is due to its appearance and its pleasant touch, or due to its ability to socially interact with humans. In the current experiment, we aimed to assess the contribution of the interactive quality to pain perception. We assessed the effect of touching the social robot PARO on mild and strong pain ratings and on stress perception, on a total of 60 healthy young participants. The robot either interacted with participants (ON group, *n* = 30) or was turned off (OFF group, *n* = 30). Touching the robot induced a decrease in mild pain ratings (compared to baseline) only in the ON group while strong pain ratings decreased similarly in both the ON and the OFF groups. The decrease in mild pain ratings in the ON group was significantly greater in participants with a higher positive perception of the interaction with PARO. We conclude that part of the effect that touching the robot has on pain stems from its interactive features.

## 1 Introduction

Positive social relationships are a core psychological need for physical and mental wellbeing (Demir et al., 2012; Diener et al., 2018; Martela and Sheldon, 2019; Soares et al., 2021). In particular, social relationship that involve social touch (i.e., stroking, hugging or holding hands) were found to reduce stress and pain perception (Goldstein et al., 2016; Morrison, 2016; Ditzen et al., 2019; Kreuder et al., 2019). The neurophysiological mechanism underlying the effect of social touch on pain are not entirely clear. Treatments that include tactile stimulation, such as massage or transcutaneous electrical nerve stimulation (TENS), activate mechanoreceptors that can inhibit the nociceptive input entering the spinal cord (Weinrich and Weinrich, 1990; Sufka and Price, 2002; Snyder and Shaparin, 2019). Beyond this segmental pain-inhibitory mechanism, affective, social touch may activate top-down pain-inhibitory mechanisms through cortical and sub-cortical brain regions (Löken and Olausson, 2010; Björnsdotter and Olausson, 2011; McGlone et al., 2014; Meijer et al., 2021). Through this mechanism, the pain-inhibitory effect depends on the social relationship and the context in which the touch occurs; For example, it was found that holding a partner’s hand during nociceptive stimulation alleviates pain significantly more than holding a stranger’s hand or an object (Master et al., 2009; Goldstein et al., 2016; Che et al., 2021). These studies highlight the connection between social interaction, touch and pain perception.

In contrast, social isolation as well as negative social interactions may lead to high levels of stress (Geva et al., 2014; Beekman et al., 2016; Helpman et al., 2017), increased pain (Stein et al., 2007; Geva et al., 2014; Geva and Defrin, 2018; Bannon et al., 2021) and health problems (Grant et al., 2009; Valtorta et al., 2016; Williamson et al., 2018). Moreover, people with chronic pain more often report interpersonal isolation, social inadequacy and alienation (Rokach et al., 2018), and thus may be in a vicious cycle, in which they do not have the opportunity to benefit from the inhibitory effect of social touch on pain perception. The COVID-19 pandemic highlighted the negative effect of social isolation. It was found that during the pandemic, the prevalence of distress and loneliness increased (Killgore et al., 2020; Rosenberg, 2020; Tull et al., 2020) and that the pandemic had a significantly negative impact on the physical and psychological health of chronic pain patients (Karos et al., 2020; Attal et al., 2021; Carrillo-de-la-Peña et al., 2021). It was also postulated that chronic pain prevalence may increase in the near future due to social distancing (Clauw et al., 2020).

A companion animal may serve as a (partial) solution for loneliness and social distancing. Previous studies demonstrate that interaction and especially petting a companion animal can provide a calming effect and increase positive emotions (Walsh, 2009; Beetz et al., 2012; Bao and Schreer, 2016; Gee et al., 2017; Charry-Sánchez et al., 2018; Young et al., 2020). However, not everyone is willing or able to take care of a pet.

Another contemporary solution for social loneliness is using social robots as companions. A social robot may look like a human or a pet, or move like one (Eizicovits et al., 2018; Kashi and Levy-Tzedek, 2018). They are designed to create a social relationship with people, and may assist users in a variety of ways, from cognitive training (Ananto and Young, 2020; Giansanti, 2021; Henschel et al., 2021; Kubota and Riek, 2021; Cohavi and Levy-Tzedek, 2022; Kubota et al., 2022) to heart and post-stroke rehabilitation (Céspedes Gómez et al., 2021; Feingold-Polak et al., 2021). Pet-like social robots have been found to create good social interactions especially if they have a soft shell (Schellin et al., 2020; Osawa et al., 2021). Shibata (2010) (Shibata, 2010) developed a social robot named PARO, a furry robotic animal in the shape of a baby seal. It has been found that interaction with PARO reduces stress and anxiety, increases positive emotions and reduces pain levels (Wada et al., 2005; Wada and Shibata, 2007; Moyle et al., 2013; Lu et al., 2021). It should be noted that the participants in all these studies were older adults with dementia or children.

We previously examined the effect of interaction with PARO on young adults (Geva et al., 2020): we tested the effect of touch as opposed to the robot’s *presence* (without any physical contact) on the perception of pain, salivary oxytocin levels and emotional state. We found that there was a reduction in pain ratings while PARO was present in the room compared to the baseline pain ratings. This effect was more pronounced when participants touched PARO compared to when it was merely present in the room with them. These results prompted the question of whether the effect that touching the robot had on pain stemmed from the social features of the robot, as in human-human and human-pet social touch, or from other sensory aspects of touch that can reduce pain, such as local (segmental) pain inhibition (Guan, 2012; Braz et al., 2014; Stachowski and Dougherty, 2021) or distraction (Johnson et al., 1998; Kucyi et al., 2013; Birnie et al., 2017).

As a first step in answering this question, we examined here the effect of touching the social robot PARO on pain and stress perception when it is turned on (with active social features), compared to when it is turned off (with no active social features).

## 2 Materials and Methods

### 2.1 Participants

Sixty healthy adults (30 females, 30 males; age: 25 ± 1.8 years) were allocated using a computer-generated simple random sampling into one of two groups: ON group (30 participants, 15 female, 15 male; 25.1 ± 1.6 years old) or OFF group (30 participants, 15 female, 15 male; 24.9 ± 2.1 years old). The participants were recruited by advertisements posted on social media. Exclusion criteria were acute or chronic pain, present or previous pathology in the arms (testing site), diseases causing potential neural damage (e.g., diabetes), systemic and mental illnesses (e.g., anxiety disorders, major depression, bipolar disorder), communication disabilities and pregnancy. Written informed consent was obtained from all participants. The experiment was approved by the institutional review board of Ben-Gurion University. All experimental procedures were performed in accordance with this ethical approval.

### 2.2 Equipment

#### 2.2.1 PARO Robot

PARO is a robot baby harp seal, manufactured by the Intelligent System Research Institute of Japan’s National Institute of Advanced Industrial Science and Technology. PARO was designed to have a calming effect and to elicit emotional responses in users (Wada et al., 2002). It has dual 32-bit processors, three microphones, twelve tactile sensors covering its fur, touch-sensitive whiskers, and a system of motors and actuators that move its limbs and body. It responds to petting by moving its tail and opening and closing its eyes. It seeks out eye contact and produces sounds intended to mimic those of a real baby seal (Wada et al., 2002).

#### 2.2.2 Thermal Stimulator

Heat pain was elicited using a Peltier-based computerized thermal stimulator (TSA II, Medoc Ltd., Ramat-Ishai, Israel), with a 3×3  cm contact probe that was attached to the ventral side of the non-dominant forearm using a Velcro band. The initial temperature of the stimulator was set to 35 °C for all the tests.

#### 2.2.3 Visual Analogue Scale

**FIGURE 1:**
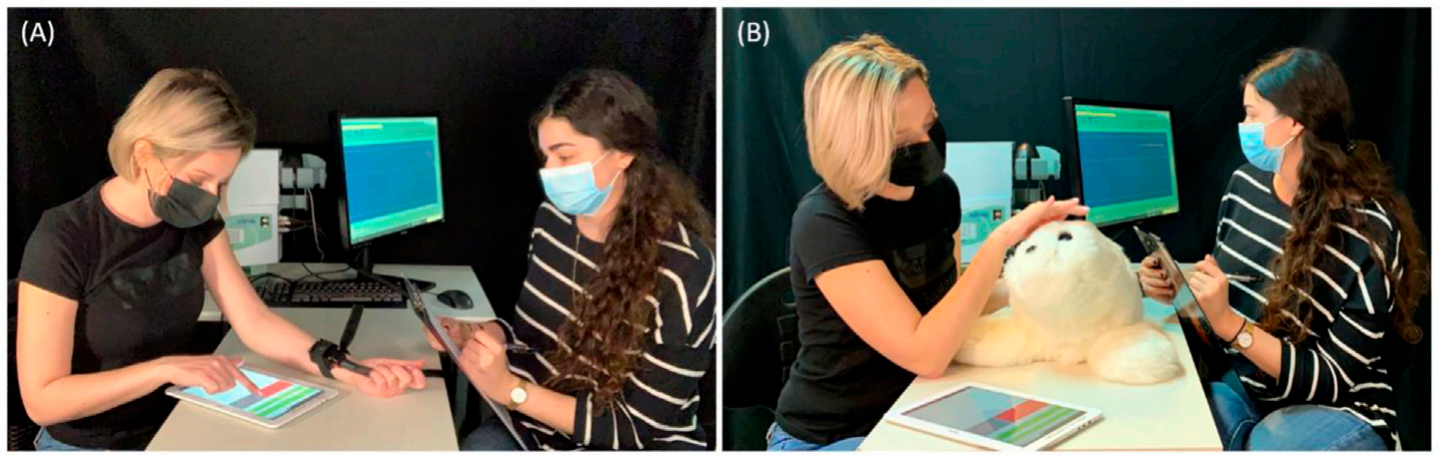
An illustration of the experimental setup. The participant (on the left) has the thermal stimulator placed on her non-dominant arm, which is placed on the table. The experimenter (on the right) administers the accurate heat stimuli, and tracks them on the screen. **(A)** Baseline condition; PARO is not present. The participant uses the custom-made VAS application to rate pain levels. In order to rate the intensity of pain, the participants were asked to slide their finger from left to right on the screen of the tablet. Sliding the finger revealed a red area that expanded as the participant slid her finger further to the right. The leftmost edge of the screen was defined to have a value of zero (no pain), and the rightmost edge was defined to have a value of 10 (the most intense pain). **(B)** Touch condition; PARO is placed on the table next to the participant, who touches it during the administration of the heat stimuli.

The visual analog scale (VAS) is a direct scaling technique, in which the response of participants is indicated by line length (Price et al., 1983). The VAS has been reported as a valid and reliable measure for rating the intensity of pain (Price et al., 1983) and emotional state (Monk, 1989). We digitally recorded participants’ pain ratings and stress levels using a custom-made computer application, installed on a tablet. Participants slid their finger on the screen, from left to right, to indicate to what extent they perceived the stimulus to be painful, or the extent to which they experienced stress (see Figure 1). For the pain ratings, the right end of the scale was defined as “no pain sensation” and the left end of the scale as “the most intense pain sensation imaginable”. For the stress-level ratings, the right end of the scale was defined as “no stress” and the left end of the scale as “the highest imaginable level of stress”. The custom-made application converted the final horizontal finger location to a number on a scale from zero to 10.

### 2.3 Experimental Setup

#### 2.3.1 Calibrating Heat-Pain Intensity

To establish which temperatures elicit in each individual sensations of mild and strong pain, participants received a series of heat stimuli in a set of calibration trials. In each calibration trial, the starting temperature of the stimulator was 35 °C, and it increased at a rate of 1 °C/sec to a target temperature. The first target temperature was 40°C. The target temperature was held for 6 sec, and participants were asked to rate the pain on the VAS application. The temperature then returned to baseline (35 °C) by an active cooling mechanism. Following a 45-sec break, the subsequent trial was initiated. An interstimulus interval of 45 s was maintained and the contact probe was moved between stimulations to prevent sensitization of the area. The target temperature was increased by 1 °C in each subsequent calibration trial until the participant reported a value of 6 (out of 10) on the VAS. The temperatures eliciting a pain-rating value of 3–4 (mild pain) and a value of 6–7 (strong pain) on the VAS were identified per participant, and used for the rest of the experiment.

#### 2.3.2 Pain Measurements

In each of the two pain measurements, the temperatures which elicited a mild pain sensation (value of 3-4 on the VAS), and a strong pain sensation (a value of 6-7 on the VAS) during the calibration phase were administered for 40 s each with an inter-stimulus interval of 2 min; the probe location was changed between stimulations. VAS pain ratings at the end of each stimulus were recorded.

#### 2.3.3 Stress State

Perceived stress was evaluated using a VAS 10-cm line with 2 anchor points at its extremes, set as “not at all” (= 0) and “the most” (= 10), and participants were asked to mark on that scale their stress level. This method was found to be reliable and valid for measuring emotional state, including stress (Barré et al., 2017).

#### 2.3.4 Familiarity Session With PARO

During the interaction with PARO, participants were asked to answer a questionnaire which encouraged them to examine PARO’s reactions. The questionnaire for each group was different, depending on whether PARO was turned ON or OFF (See Supplementary Materials S1, S2 for the respective full questionnaires). The goal of asking participants to fill out these questionnaires was to ensure that they spent the session touching PARO and actively engaging with it. The OFF-group participants were not told that the robot had any robotic features.

#### 2.3.5 Perceptions of the Interaction With PARO

At the end of the experiment we evaluated ON-group participants’ perceptions regarding the interaction with PARO using a 5-item custom-made questionnaire, to which participants responded using a 10-cm VAS line with 2 anchor points at its extremes, set to “not at all” (= 0) and “the most” (= 10). The questions were: 1) Is it pleasant to touch PARO? 2) Did you feel good with PARO? 3) Did you feel you could communicate with PARO? 4) Did PARO help to reduce pain? 5) Would you like to meet PARO again? The OFF-group participants were asked: Is it pleasant to touch PARO?

### 2.4 Procedure

**FIGURE 2:**
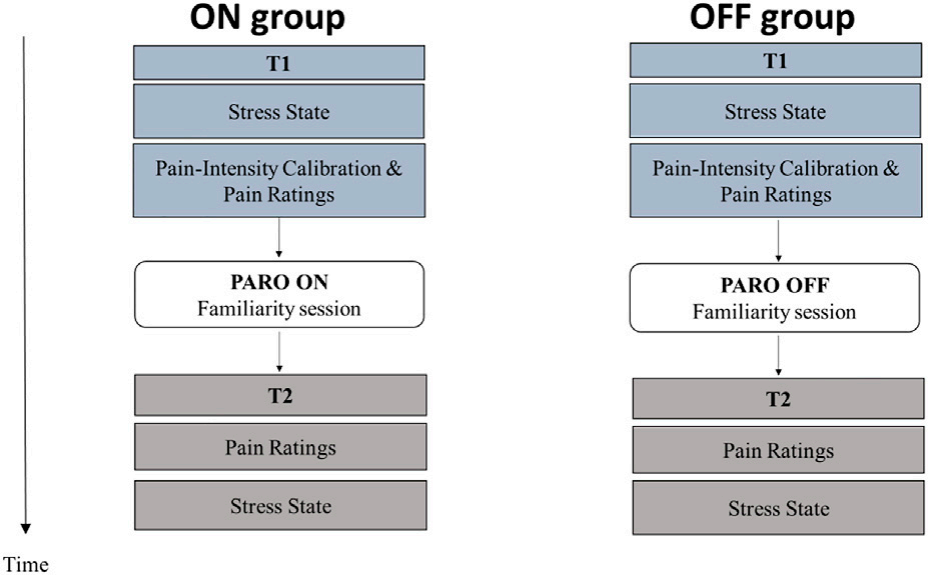
Flow chart of the experimental design.

Each participant was invited to a single testing session that lasted approximately 45 min. The participants were instructed to avoid physical exercise for 1 hour before testing. Upon arrival, participants were divided semi-randomly to either the ON group or the OFF group, to ensure equal numbers of males and females in each group. Testing took place in a quiet room. The temperature was maintained at 25 °C and the participant sat in a comfortable chair. Five minutes after arrival, the first stress-state rating was obtained, followed by the pain-intensity calibration and the first pain measurements (T1). Immediately after that, participants in both groups spent 5 min in a structured familiarity session with PARO. The participants in both groups were encouraged to touch and stroke PARO throughout the familiarity session. They then underwent the pain measurements while touching PARO, which was either in interactive mode (ON), or in inactive mode (OFF). They placed the dominant hand on PARO while receiving the sensory stimuli on their non-dominant inner forearm. Then the participants rated their perceptions of the contact with PARO and finally, both groups underwent the second stress state rating (T2) (Figure 2).

### 2.5 Data Analysis

Data were analyzed using IBM SPSS statistic software version 27. All data underwent Kolmogorov-Smirnov analysis for normality of distribution. Continuous variables are described as means ± standard deviation. Parametric and nonparametric ANOVA (Kruskal-Wallis analysis) of variance with corrected post-hoc Bonferroni tests were used to evaluate the effect of experimental condition (T1/T2) and of group (ON/OFF) on pain ratings. Effects size of post-hoc comparisons were evaluated with Cohen’s d. Differences in the outcome measures between T1 and T2 conditions (delta) were calculated by subtracting the former from the latter. Correlations between pairs of variables were calculated with Pearson’s r; *p* < 0.05 was considered significant. The Bonferroni correction was applied to multiple comparisons, where needed.

## 3 Results

### 3.1 The Effect of the Interaction With PARO on the Participants’ Perceived Stress

**FIGURE 3:**
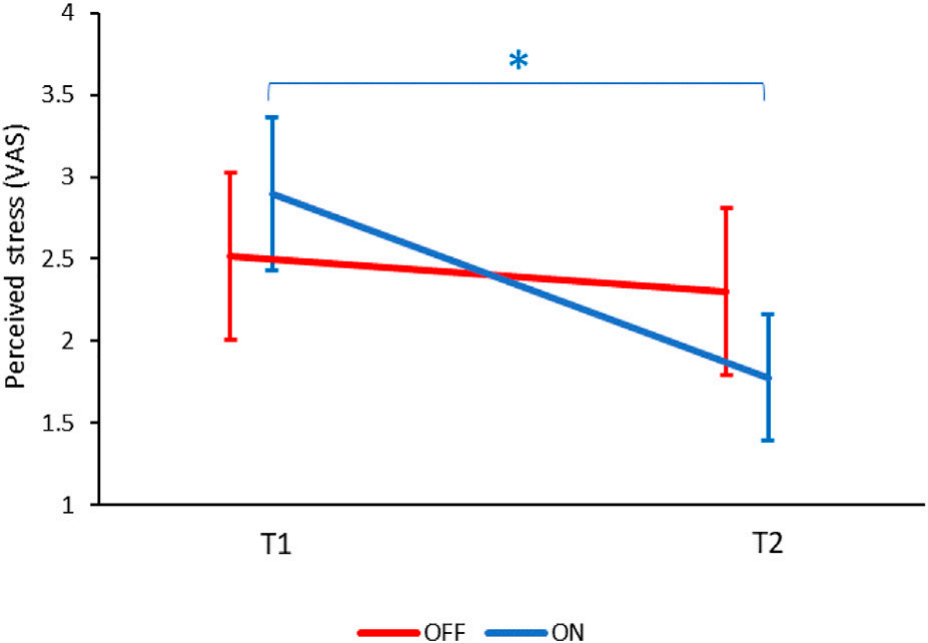
The change in perceived stress between BASELINE (T1) and TOUCH (T2) in the ON and OFF groups. Only in the ON group perceived stress decreased from T1 to T2 (*p < 0.05). Values denote mean visual-analogue scale (VAS) ratings (0–10) ± SEM. Results from the ON group are marked in blue, and results from the OFF group are marked in red. T1—before encountering PARO, T2—while touching PARO.

When tested across all participants, stress levels did not change significantly from T1 (BASELINE condition) to T2 (TOUCH condition) [F (1,60) = 3.8, *p* = 0.05]. There was no significant effect of the group (ON/OFF) [F (1,60) = 0.2, *p* = 0.9) and no interaction effect of condition*group [f (1,60) = 1.8, *p* = 0.2]. No gender effects were found. However, post hoc tests revealed that in the ON group there was a significant reduction in stress ratings from T1 (2.9 ± 2.5) to T2 (1.8 ± 2.1, *p* < 0.05), while in the OFF group there was no significant reduction from T1 (2.5 ± 2.7) to T2 (2.3 ± 2.7, *p* = 0.3; see Figure 3).

### 3.2 The Effect of the Interaction With PARO on Pain Perception

#### 3.2.1 Mild Pain

**FIGURE 4:**
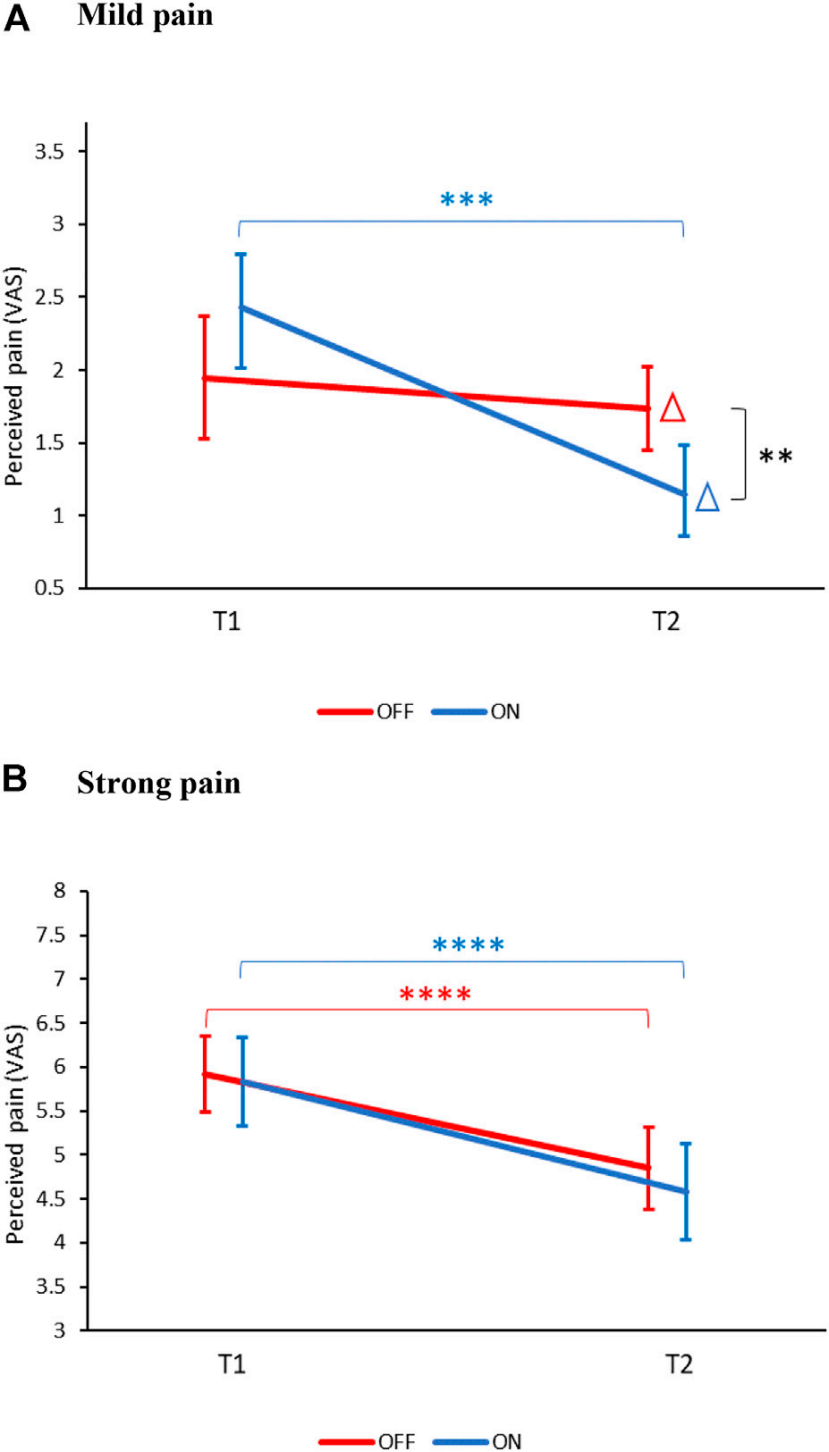
Changes in pain perception between BASELINE (T1) and TOUCH (T2). **(A)** Mild pain: The change in pain ratings (marked with a Δ) from T1 to T2 was significantly different between the ON (blue) and OFF (red) groups (**p < 0.01). Only in the ON group, pain ratings decreased significantly at T2 compared to T1 (***p < 0.001). **(B)** Strong pain: In both the ON and the OFF groups, pain ratings decreased at T2 compared to T1 (***p < 0.0001). Values denote mean visual-analogue scale (VAS) ratings (0–10) ± SEM. T1—before encountering PARO, T2—while touching PARO.

Figure 4A presents the change in mild pain VAS scores from baseline (T1) to touching PARO (T2). A significant effect of condition (T1/T2) [F (1,60) = 11.12, *p* < 0.01) was found, suggesting a change in pain ratings between T1 to T2. The effect of group (ON/OFF) was not significant (F (1,60) = 0.1, *p* = 0.90). However, the interaction effect of condition*group was significant (F (1,60) = 5.8, *p* < 0.05), indicating that the magnitude of pain inhibition was different between ON and OFF groups. No gender effects were found.

Post hoc tests revealed that there was no significant difference in pain ratings at T1 between the ON (2.0 ± 1.7) and the OFF (2.4 ± 1.9, *p* = 0.19) groups. However, only in the ON group there was a significant decrease in pain ratings from T1 to T2 (delta of 1.3 ± 1.8 VAS units, *p* < 0.01) while no significant difference was found in the OFF group (delta of 0.2 ± 1.6 VAS units, *p* = 0.24).

In the ON group there were negative correlations between participants’ mild pain ratings during T2 and their perception of the interaction with PARO: their willingness to meet PARO again (r = −0.4, *p* < 0.05) and their feeling that PARO helped alleviating pain (r = −0.5, *p* < 0.01). That is, the lower their pain ratings during the interaction with PARO, the higher their willingness to meet PARO again and their feeling that PARO helped to alleviate pain. In addition, there were also correlations between the decrease in pain ratings (from T1 to T2) and the participants’ perceptions of the interaction with PARO: pleasant touch (r = 0.3, *p* < 0.05) and willingness to meet PARO again (r = 0.5, *p* < 0.01).

#### 3.2.2 Strong Pain

Figure 4B presents the change in strong-pain VAS scores from baseline (T1) to touching PARO (T2). A significant effect of condition (T1/T2) [F (1,60) = 30.5, *p* < 0.0001) was found, suggesting a change in pain ratings from T1 to T2. The effect of group (ON/OFF) (F (1,60) = 0.8) and the interaction condition*group (F (1,60) = 0.20) were not significant, suggesting that the change in pain ratings from T1 to T2 was similar in the ON and the OFF groups. No gender effects were found.

We found the following significant correlations in the ON group: 1) between participants’ strong pain ratings at T2 and the perception of pleasant touch (r = 0.3, *p* < 0.05); 2) between the decrease in strong pain ratings (from T1 to T2) and the perception of good feeling with PARO (r = 0.4, *p* < 0.05); and their willingness to meet PARO again (r = 0.4, *p* < 0.01).

In the OFF group we found a negative correlation between the decrease in strong pain ratings from T1 to T2 and their perceived stress at T1 (r = −0.3, *p* < 0.05). That is, the lower their perceived stress at baseline, the greater the reduction in pain ratings from baseline to the touch condition.

### 3.3 Perceptions of the Interaction With PARO

All participants were asked how pleasant was the sensation of touching PARO. There was no statistically significant difference in the ratings of pleasantness of touch between the ON group (7.0 ± 2.5) and the OFF group (7.8 ± 1.6; *p* = 0.06).

The participants in the ON group gave high ratings when asked how good they felt in the presence of PARO (7.1 ± 2.8). They gave intermediate ratings when asked about their willingness to meet PARO again (5.4 ± 2.7), about their perceived ability to communicate with PARO (4.2 ± 3.0), and whether they thought PARO helped to reduce pain (4.1 ± 3.0).

To further investigate the effect of the interaction with PARO on pain perception, we divided the participants according to their mean ratings of pleasantness of touch, perceived ability to communicate with PARO and willingness to meet PARO again, as detailed below.

#### 3.3.1 Pleasantness of Touch

We divided the participants in each group according to the pleasantness-of-touch ratings into high pleasantness of touch (HPT) and low pleasantness of touch (LPT). The division was made using the median value of participants’ perceived pleasantness of touching PARO (7.0 in the ON group, 7.8 in the OFF group). Among the ON group, the mean pleasantness of touch was 9.1 ± 1.0 for HPT (*n* = 15) and 4.8 ± 1.6 for LPT (*n* = 15) (*p* < 0.0001). Among the OFF group, the mean pleasantness of touch was 9.1 ± 0.7 for HPT (*n* = 14) and 6.7 ± 1.2 for LPT (*n* = 16) (*p* < 0.0001).

**FIGURE 5:**
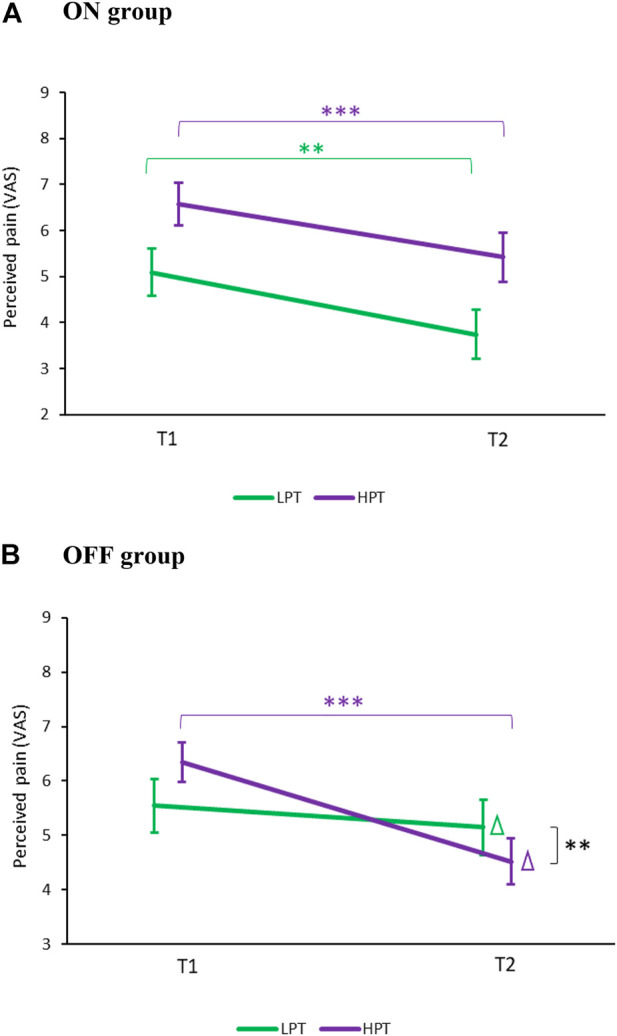
Differences in strong-pain ratings between participants who gave high vs. low ratings to the pleasantness of touching PARO. **(A)** Within the ON group there was a significant decrease in pain ratings from T1 to T2 both in HPT (***p < 0.001, purple) and LPT (**p < 0.01, green). **(B)** Within the OFF group there was a significant decrease in pain ratings from T1 to T2 only in HPT (***p < 0.001). The change in pain ratings from T1 to T2 (marked with a Δ) was significantly different between the HPT and LPT sub-groups (**p < 0.01). Values denote mean visual-analogue scale (VAS) ratings (0–10) ± SEM. T1—before encountering PARO, T2—while touching PARO. HPT—participants who gave above-median ratings to the pleasantness of touching PARO. LPT—participants who gave below-median ratings to the pleasantness of touching PARO.

Figure 5 presents the strong-pain ratings for T1 and T2 by participants in the HPT and LPT sub-groups within the ON and the OFF groups. At T1, there was no difference between pain ratings by participants in the HPT and the LPT groups in either the ON (6.6 ± 2.5 and 5.1 ± 2.8 respectively, *p* = 0.07) or the OFF (6.3 ± 2.0 and 5.5 ± 2.6 respectively, *p* = 0.18). Only in the OFF group, there was a significantly greater decrease in pain ratings within the HPT sub-group (delta of 1.8 ± 1.5) compared to the LPT subgroup (delta of 0.4 ± 1.5, *p* < 0.01). For mild pain ratings, there were no significant differences between HPT and LPT both in OFF and ON groups.

#### 3.3.2 Communication With PARO

**FIGURE 6:**
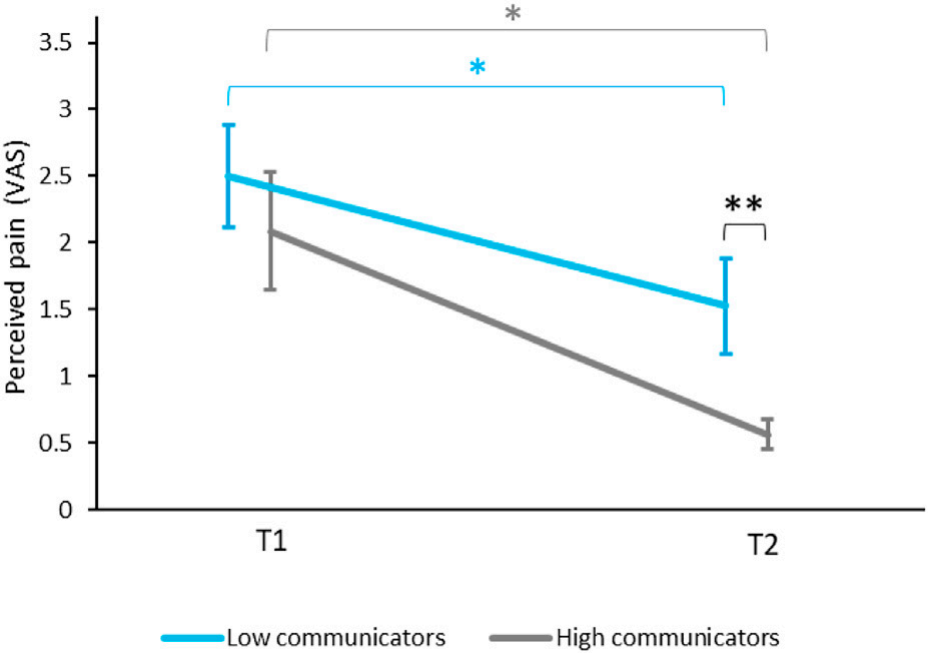
Differences in mild-pain ratings between high communicators (HC) and low communicators (LC). Pain ratings decreased at T2 compared to T1 in both the HC (*p < 0.05, gray) and the LC groups (*p < 0.05, blue). However, at T2 pain ratings by HC were significantly lower than ratings by LC (**p < 0.01). Values denote mean visual-analogue scale (VAS) ratings (0–10) ± SEM. T1—before encountering PARO, T2—while touching PARO. HC—participants who gave above-median ratings to their ability to communicate with PARO. LC—participants who gave below-median ratings to their ability to communicate with PARO.

We divided the participants in the ON group into high communicators (HC) and low communicators (LC). The division into the two groups was made using the median value of participants’ perceived ability to communicate with PARO (3.8). The mean communication rating was 6.8 ± 1.8 for HC (*n* = 14) and 1.5 ± 1.0 for LC (*n* = 15) (*p* < 0.0001). Figure 6 presents the mild-pain ratings for T1 and T2 conditions by High and Low communicators. There was no significant difference in pain ratings at T1 between high (2.1 ± 2.4) and low communicators (2.5 ± 2.1, *p* = 0.31). However, at T2 pain ratings by high communicators (0.5 ± 0.6) were significantly lower than ratings by low communicators (1.5 ± 1.9, *p* < 0.05). One participant was excluded from the HC analysis due to irregular values in T2 pain ratings (4.7 standard deviations from the mean).

#### 3.3.3 Meeting PARO Again

**FIGURE 7:**
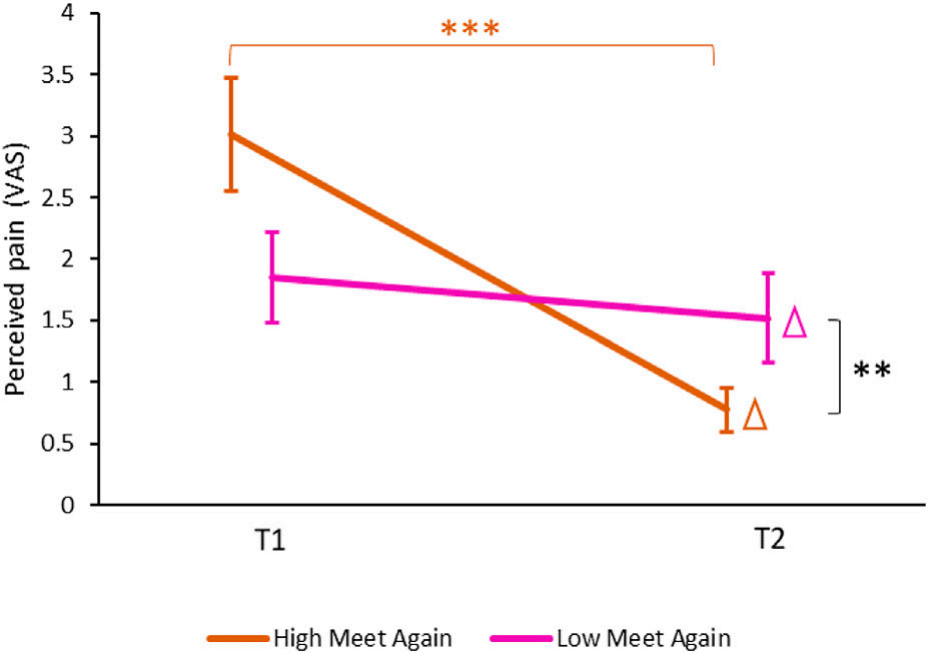
Differences in pain ratings between HMA and LMA. Only in the HMA sub-group (brown) there was a significant decrease in pain ratings at T2 compared to T1 (***p < 0.001). The change in pain ratings from T1 to T2 (marked with a Δ) was significantly different between the HMA and the LMA (magenta) sub-groups (**p < 0.01). Values denote mean visual-analogue scale (VAS) ratings (0–10) ± SEM. T1—before encountering PARO, T2—while touching PARO. HMA—participants who gave above-median ratings to their willingness to meet PARO again. LMA—participants who gave below-median ratings to their willingness to meet PARO again.

We divided the participants in the ON group into high willingness to meet PARO again (HMA) and low willingness to meet PARO again (LMA). The division into the two groups was made using the median value of the response to the question: “to what extent would you want to meet PARO again?” (5.7 ± 3.9). The mean ratings were 8.8 ± 1.4 for the HMA group (*n* = 15) and 1.9 ± 2.1 for the LMA group (*n* = 15) (*p* < 0.0001). Figure 7 presents the mild pain ratings for T1 and T2 conditions in HMA and LMA. There was no significant difference in pain ratings at T1 between high (3.0 ± 2.5) and low MA (1.9 ± 2.0, *p* = 0.09). However, the decrease in pain ratings from T1 to T2 was significantly greater among HMA (delta of 2.2 ± 2.1) compared to LMA (delta of 0.3 ± 0.7, *p* < 0.01).

## 4 Discussion

We found that touching the social robot PARO reduced pain ratings for both mild and strong heat stimuli. The reduction in mild pain ratings was significant only in the group that touched PARO when it was turned ON. The reduction in pain ratings in this group correlated with the participants’ positive perceptions of the interaction with PARO in response to both mild- and strong-pain stimuli: those who enjoyed the interaction with it more experienced a greater reduction in perceived pain. Moreover, within the ON group, participants who perceived themselves as communicating well with PARO, or who wished to meet it again, demonstrated a significantly greater decrease in pain during the social touch. Within the OFF group, however, participants who perceived PARO as more pleasant to touch demonstrated a significantly greater decrease in pain while touching PARO.

### 4.1 The Effect of Social-Touching PARO on Stress

Only the ON-group participants reported a decrease in stress levels after the interaction with PARO which was found to be significant.

Affective social touch among humans is known to reduce stress and anxiety (Ditzen et al., 2007; Burleson and Davis, 2013; Morrison, 2016; Ditzen et al., 2019). Tactile information of pleasant touch (such as stroking) is processed in the insula, which down-regulates the stress response via amygdalar inhibition (Eckstein et al., 2020). It was suggested that social touch can act as a social signal for safety, and thus reduce the degree of anxiety (Morrison, 2016; Eckstein et al., 2020) and regulate stress (Morrison, 2016; Fotopoulou et al., 2022). These beneficial effects of social touch were found both for the toucher and the receiver of the touch (Schirmer et al., 2022). Especially during exposure to acute or chronic stressors, social touch may act as a stress buffer and reduce the physiological and behavioral stress response (Morrison, 2016; Dreisoerner et al., 2021). Petting animals was similarly found to reduce stress and anxiety. Shiloh et al. (Shiloh et al., 2003) randomly assigned participants to petting a rabbit, a turtle, a toy rabbit, a toy turtle or to a control group after a stressful situation. The authors found that petting an animal, but not a matched doll, reduced state-anxiety. The anxiolytic effect was observed both for petting a rabbit and a turtle, demonstrating that social touch rather than the texture of the object produced the effect. Interestingly, the anxiolytic effect was not restricted to animal lovers. Martos-Montes et al. (Martos-Montes et al., 2019) similarly found that petting a dog after a psychosocial stress task, reduced the levels of anxiety and heart rate compared to petting a toy dog.

Previous studies demonstrate that a similar effect on emotions can be achieved by human-robot interactions (HRI) (though not necessarily to the same extent). For example, several studies demonstrated that HRI can increase positive emotions among healthy participants (Crossman et al., 2018; Geva et al., 2020; Hoffmann and Krämer, 2021). HRI was also found to reduce student stress during final exams (Edwards et al., 2020), reduce stress and anxiety among hospitalized children undergoing a medical procedure (Beran et al., 2013; Okita, 2013; Beran et al., 2015; Ruocco et al., 2019; Trost et al., 2019; Trost et al., 2020; Smakman et al., 2021) and among older adults living in senior healthcare settings (Góngora Alonso et al., 2019; Pu et al., 2019). It is important to note that all the mentioned studies did not report the role of plain touch compared to social touch in HRI.

We found three previous studies that compared how an interaction with a robot affects emotions differently from a similar-looking doll. Tanaka et al. (Tanaka et al., 2012) randomly assigned older women to receive either a social robot, designed to resemble a 3-year-old boy, or a control robot (inactive) with the same appearance; the object (active robot or control robot) was placed in their homes for a period of 8 weeks. In the social-robot group, there was a significant decrease in salivary cortisol levels (a measure of the physiological stress response) while no change was observed in the control-robot group (Tanaka et al., 2012). Kidd et al. (Kidd et al., 2006) investigated the interaction with PARO turned ON vs. turned OFF among older individuals living in a nursery home. The authors conclude that social dimensions significantly improve the benefits of the interaction with PARO (Kidd et al., 2006). Crossman et al. (Crossman et al., 2018) compared the effect that social touching PARO had on emotions in its ON vs. its OFF state. The authors reported that positive emotions increased to a greater extent when participants touched it in the ON state, compared to the OFF state. They concluded that experience with the robot, rather than some other appealing feature of the robot (e.g., that it is soft), produced the increase in the positive affect. Taken together, it appears that interaction with a social robot is effective among all age groups in creating positive emotions as well as in reducing stress during stressful situations (Edwards et al., 2020), including during hospitalization [e.g., 86]. In accordance with these previous studies, we show here that *social* touching a robot is more effective for stress reduction than touch alone.

### 4.2 The Effect of the Interaction with PARO on Pain Perception

In the mild-pain stimuli, we found a significant reduction in pain perception only in the group that touched PARO in the ON mode, and not in the group that touched PARO in the OFF mode. In the strong-pain stimuli, pain perception decreased in both the ON and the OFF groups. These results suggest that human-robot social touch has a greater effect on pain inhibition than plain touch.

Our findings are in line with previous studies which found that human-robot social interaction is effective in diminishing clinical pain. This effect was found in studies where the HRI was without any physical contact with the robot (Beran et al., 2013; Yasemin et al., 2016; Ruocco et al., 2019; Trost et al., 2020; Smakman et al., 2021) as well as in studies where the HRI involved touch (Such as hugging the robot and/or stroking it) (Tanaka et al., 2012; Eskander et al., 2013; Okita, 2013; Pu et al., 2019). In our previous experiment (Geva et al., 2020) we demonstrated that touching a social robot is more effective for pain reduction than social interaction with the robot that does not include physical contact.

There are several possible explanations for the analgesic effect of social touching PARO. First, the effect of touch on pain perception can be attributed to the discriminative properties of touch: it is known that tactile information, transmitted by A beta fibers to the spinal cord, can suppress nociceptive information in the same segment by secreting inhibitory neurotransmitters in the posterior horn of the spinal cord (Salter and Henry, 1990a; Salter and Henry, 1990b; Bardoni et al., 2013; Mendell, 2014; West et al., 2015). It was recently found that pleasant touch, such as stroking, is transmitted by tactile C-fibers and can also suppress nociceptive information in the same segment (Liljencrantz et al., 2016; Fidanza et al., 2021). However, since the pain modulation via this mechanism is, as far as is known today, segmental (Mancini et al., 19362015; Stachowski and Dougherty, 2021), it cannot explain the decrease in pain in an area far from the pleasant touch. Therefore, it does not give a sufficient answer to the decrease in pain in the present study where touching PARO was done with the opposite hand to where the nociceptive stimuli were applied.

Another conceivable explanation is that touching PARO during the nociceptive stimulus distracted participants from the painful stimuli. It is already known that a cognitive distraction can lead to a decrease in pain (Buhle and Wager, 2010; Kucyi et al., 2013; Loreto-Quijada et al., 2014; Gupta et al., 2018; Gates et al., 2020). However, a number of studies suggest that the effect of social touch on pain does not stem primarily from distraction. For example, gentle skin stroking alleviated pain ratings while controlling for distraction through temporally separated stimuli (Liljencrantz et al., 2017). In another study, women received nociceptive thermal stimulation in different social and non-social conditions that included holding the hand of the partner and holding an object (a squeeze ball). In order to assess whether participants were more distracted in one condition, the participants were asked to press the space bar on computer-generated beeps that were infrequently and randomly emitted throughout the study. The results reveal that the reaction time was similar in all conditions, and thus the social support condition was not confounded with distraction (Master et al., 2009). In addition, women receiving social touch (hand holding) had reduced perceived pain and attenuated functional magnetic resonance imaging activity in pain-processing brain networks which were distinct from the functional network associated with cognitive distraction (López-Solà et al., 2019). The above studies focused on human-human social touch. Less is known about the effect of distraction in human-robot social touch. To study the effect of distraction, one recent pilot study compared the effect of interaction with social assistive humanoid robot programmed to distraction vs. empathy on pain and fear ratings among hospitalized children during insertion of peripheral intravenous (IV) catheter (Trost et al., 2020). Mean pain and fear scores were lowest in the empathy group immediately before and after IV placement. In addition, children were more likely to attribute characteristics of empathy to the robot programmed for the empathic condition. The results of the current study also further emphasize the involvement of pain inhibitory mechanisms other than distraction in human-robot social interaction. First, the very presence of PARO, even when it was turned OFF also constitutes a distraction, and yet the decrease in mild pain was greater during social touching PARO than when touching it in its OFF mode. Additionally, within the ON group there was a positive correlation between the decrease in pain ratings and the perception of feeling good during the social touch. Moreover, the division of the ON group according to their perceptions of the interaction with PARO verified that participants who perceived themselves as highly able to communicate with PARO and participants who were willing to meet PARO again exhibited significantly greater pain inhibition during social touch compared to low communicators and those less willing to meet PARO again—results which echo those we documented in our previous work (Geva et al., 2020). These findings highlight the role of positive social interactions in pain inhibition during human-robot contact.

A possible underlying mechanism for the pain inhibition during social touch is the effect of stress reduction on pain. It is already well known that there are bidirectional neural interactions between pain and stress (Sawicki et al., 2021). During acute social stress, fear and anxiety, the amygdala activates the dorsomedial hypothalamic nucleus (DMH) which, in turn, may suppress pain-inhibitory pathways through the rostro ventral medulla (RVM) (Davis, 1992; Roozendaal et al., 2009; Tovote et al., 2015; Kataoka et al., 2020). Accordingly, a number of studies in which a model of negative social relationships was used to elicit acute stress, found that the stress manipulation induced pain amplification (Geva et al., 2014; Geva and Defrin, 2018; Mertens et al., 2020).

In addition, previous studies demonstrated that interventions aimed at reducing stress, such as relaxation techniques and mindfulness lead to pain reduction (Heidarigorji et al., 2014; Feuille and Pargament, 2015; Hilton et al., 2017; Nelson et al., 2017; Case et al., 2021). Social, affective touch can induce relaxation and stress reduction which is attributed to insular suppression of the amygdala (Ellingsen et al., 2016; Eckstein et al., 2020). Interaction with PARO was found to induce a sense of calm and positive feelings (Pu et al., 2019; Geva et al., 2020). Taken together, we suggest that social-touching PARO induced positive feelings, reduced stress and thus had a greater effect on pain inhibition then touching an inanimate object. The reduction in stress ratings while social-touching PARO, but not when it is turned OFF, supports this notion.

An additional explanation of the effect of social touching PARO on pain perception is activation of the opioid system. Endogenous opioids are key players in acute pain inhibition (Roques et al., 2012; Pacheco et al., 2014; Zhang et al., 2020). Pharmacological studies in non-human primates (Meller et al., 1980; Manduca et al., 2014) as well as preliminary results among humans (Massaccesi et al., 2021), indicate that administration of µ-opioid antagonists increase motivation for social contact and affiliative touch behaviors. In line with this, increased μ‐opioid receptor (MOR) availability among humans was associated with social attachment (Nummenmaa et al., 2015). In a PET study in men, social touch increased MOR availability in the anterior cingulate cortex (ACC), insula and other brain regions associated with socioemotional processes (Nummenmaa et al., 2016). In recent years a growing number of reviews suggest that endogenous opioids serve as important neurotransmitters that enable social touch and positive social connections (Ellingsen et al., 2016; Morrison, 2016; Su and Su, 2018; Jablonski, 2021). Accordingly, it is conceivable that social touching PARO increased secretion of endogenous opioids and MOR availability which enabled a stronger pain inhibition. It should be noted that oxytocin is another important neurotransmitter involved in social touch, but its role in pain inhibition is still not completely clear. In our previous study, we found a decrease in salivary oxytocin during interaction with PARO (Geva et al., 2020). This finding is in contrast to the increase in oxytocin secretion during social contact between humans as well as non-human primates (Feldman et al., 2010; Feldman et al., 2011; Tang et al., 2020), and may indicate that human-robot social touch affects socioemotional brain processes differently.

The effect of touching PARO on pain was found to be different between mild vs. strong pain. For mild pain, there was a decrease in pain ratings only in the social-touch (ON) group, whereas for strong pain, there was a decrease in both the ON and the OFF groups. The decrease in strong pain ratings among the OFF group can be attributed to the pleasantness of touching PARO. We found that only among the OFF-group participants, the degree of *pleasantness of touch* predicted pain inhibition. Namely, only participants who perceived PARO as very pleasant to touch (mean rating of 9 out of 10) demonstrated pain inhibition during touch compared to baseline pain ratings. Whereas among the ON-group participants, there was no difference in pain inhibition magnitude between participants with high vs. low ratings for pleasantness of touch. A possible mechanism underlying these findings is a decrease in stress and anxiety during non-social pleasant touch. Animal studies demonstrate that passive gentle stimuli significantly reduce stress response among rat pups separated from the dam (Kuhn et al., 1990; van Oers et al., 1998). More recently, stimulation of C-tactile afferents was found to induce positive reinforcing and anxiolytic behavior (Vrontou et al., 2013). Similarly, among humans, optimal velocity of non-social soft brushing reduced the physiological stress response among infants (Fairhurst et al., 2014) and adults (Triscoli et al., 2017). Taken together, we postulate that the pleasant non-social touch produced by stroking PARO while off, mirrored the effect of gentle social touch on stress and anxiety during noxious stimuli and thus inhibits pain. Supporting this notion, we found a correlation between the decrease in pain ratings and perceived stress only in the OFF group, whereas in the ON group we found a correlation between the decrease in pain ratings and feeling good in the presence of *PARO*. It is possible that when stroking an inanimate object, participants are more focused in the pleasantness of its touch, whereas while stroking a social robot, participants are more focused on its social characteristics. Thus, it can be speculated that the reduction in pain stemmed from different pain-inhibitory mechanisms in each group: social touch in the ON group and stress reduction in the OFF group. Notably, for less intense pain we found a clear advantage of social touching PARO for pain relief.

### 4.3 Limitations and Future Directions

Below we list some of the study’s limitations, and future directions that might be inspired by the current work:

1) The different features of PARO when it is turned ON and OFF dictated the use of one out of five questions about the perception of the interaction with PARO in the OFF group. However, it would be instructive to conduct a follow-up experiment in which identical questions are administered to both groups, and the answers compared.
2) Participants were not monitored during the familiarity session with PARO. It is possible that different interaction patterns emerged in the high-communicators vs. the low-communicators subgroups, as well as in the participants with high willingness to meet PARO again vs. those with low willingness to meet it again; E.g., perhaps HCs and HMAs tended to stroke PARO more times than the other subgroups—which may, in turn, have affected their perception of pain and stress. It would be of interest to track the pattern of interaction in future research, to establish whether there is a connection between this pattern and the beneficial effect of the interaction. Should such a pattern emerge, it could be used as a model, according to which guided interactions with PARO can be designed to transition individuals from the LC and LMA subgroups into the HC and HMA subgroups, assuming they will then gain more by the interaction.
3) In a previous study, we documented the effects of touching PARO, while in the ON state, on levels of salivary oxytocin. It would be interesting to track the profile of salivary cortisol levels before, during, and after an interaction with PARO.
4) The study took place during the COVID-19 pandemic. The experimenter and the participants were wearing a mask throughout the experiment. The potential effect of wearing a mask on experimentally induced pain ratings and stress should be explored in future work.
5) The effect of touching PARO on pain ratings and stress may change over time: the beneficial effect observed here may be enhanced or diminished, and the direction of the change might be different for high communicators vs. low communicators. Charting these patterns over a long-term interaction will be an important step in understanding how HRI may be helpful in pain and stress reduction in the long term.
6) Our results suggest that PARO, an animal-like robot, is helpful in reducing the perception of pain. It would be of interest to explore the effects of a human-like robot in this context.

## 4.4 Conclusion

This study demonstrates that touching the social robot PARO when it is turned ON is more effective in reducing stress and mild pain levels than touching it while it is turned OFF. Participants with high perceived ability to communicate with PARO and with high willingness to meet PARO again exhibited greater pain inhibition during social touching PARO. Participants who perceived PARO as very pleasant to touch exhibited greater pain inhibition when touching PARO while it was turned OFF. These finding may add insight to the effect of social touch on pain perception and may have clinical implications for using social robots in the treatment of acute and chronic pain. This is of special importance during the COVID-19 pandemic, which led to a requirement for social distancing.

## Data Availability

The raw data supporting the conclusions of this article will be made available by the authors, without undue reservation.
