# Supplementary material for: Interaction Matters: The Effect of Touching the Social Robot PARO on Pain and Stress is Stronger When Turned ON vs. OFF
Source: Front Robot AI. 2022 Jul 8;9:926185. doi: 10.3389/frobt.2022.926185 (PMC9305613; doi:10.3389/frobt.2022.926185)
Supplement: Supplementary file 1 [file DataSheet1.docx]

**Supplementary Materials**

Questionnaire #1 – Getting to know PARO – ON group

Please indicate PARO'S reaction to the following:

When I pet its back several times ____________________

When I pet its face several times ____________________

When I call it by its name several times ________________

Please mark whether you observed PARO perform any of the following:

Move its back flippers ⃝ Yes ⃝ No

Open and close its eyes ⃝Yes ⃝ No

Make sounds ⃝ Yes ⃝ No

Do you think PARO is heavy? (please mark one response)

- Yes, it is heavy and it is fine.
- Yes, it is heavy and it is bothersome.
- No, it is not heavy and it is fine.
- No, it is not heavy and it is strange.

**Questionnaire S1. Getting to know PARO - ON group.** This questionnaire was administered to the ON-group participants during the 5-minute interaction session with PARO, prior to conducting the pain measurements while touching PARO.

Questionnaire #2 – Getting to know PARO – OFF group

Answer the following questions about PARO:

Please pet PARO and mark on the following line to what extent do you think it is pleasant to touch PARO

This question was answered using a 10-cm VAS line with 2 anchor points at its extremes, set to “not pleasant at all” (= 0) and “very pleasant ” (= 10)

What's the difference between PARO'S front and back flippers? ______

Which area is more pleasant to pet in PARO?

- The head.
- The belly.
- Other – Which area?

Do you think PARO is heavy? (please mark one response)

- Yes, it is heavy and it is fine.
- Yes, it is heavy and it is bothersome.
- No, it is not heavy and it is fine.
- No, it is not heavy and it is strange.

If you had to find PARO a new name, how would you call it?________________

How many lashes does PARO have in both eyes together?___________

What do you think is the age of PARO?_______________

What do you think about PARO?

**Questionnaire S2. Getting to know PARO - OFF group.** This questionnaire was administered to the OFF-group participants during the 5-minute familiarity session with PARO, prior to conducting the pain measurements while touching PARO.
